# Directed differentiation of human pluripotent stem cells into epidermal keratinocyte-like cells

**DOI:** 10.1016/j.xpro.2022.101613

**Published:** 2022-08-08

**Authors:** Gowher Ali, Essam M. Abdelalim

**Affiliations:** 1 Diabetes Research Center, Qatar Biomedical Research Institute (QBRI), Hamad Bin Khalifa University (HBKU), Qatar Foundation (QF), PO Box 34110, Doha, Qatar; 2 College of Health and Life Sciences, Hamad Bin Khalifa University (HBKU), Qatar Foundation, Education City, Doha, Qatar

**Keywords:** Cell culture, Cell differentiation, Stem cells

## Abstract

The human pluripotent stem cell (hPSC) differentiation has allowed for the generation of *in vitro* models to study human diseases in a dish. This protocol describes the generation of keratinocyte-like cells from hPSCs in chemically defined media. Treating hPSCs with retinoic acid and BMP4 induced the generation of keratinocyte progenitors, which further differentiated into mature keratinocytes in the presence of calcium. The keratinocytes generated with this protocol could be used to study keratinocyte biology, drug screening, and skin-related diseases.

For complete details on the use and execution of this protocol, please refer to Ali et al. (2020).

## Highlights

• Protocol for differentiation of pluripotent stem cells into epidermal keratinocytes
• Step-by-step guide for evaluating the keratinocyte differentiation efficiency
• Retinoic acid (RA) and BMP4 induce keratinocyte progenitor differentiation
• Addition of calcium enhances the maturation of epidermal keratinocyte

## Abstract (editor highlights)

Publisher’s note: Undertaking any experimental protocol requires adherence to local institutional guidelines for laboratory safety and ethics.

## Graphical abstract

**Figure:**
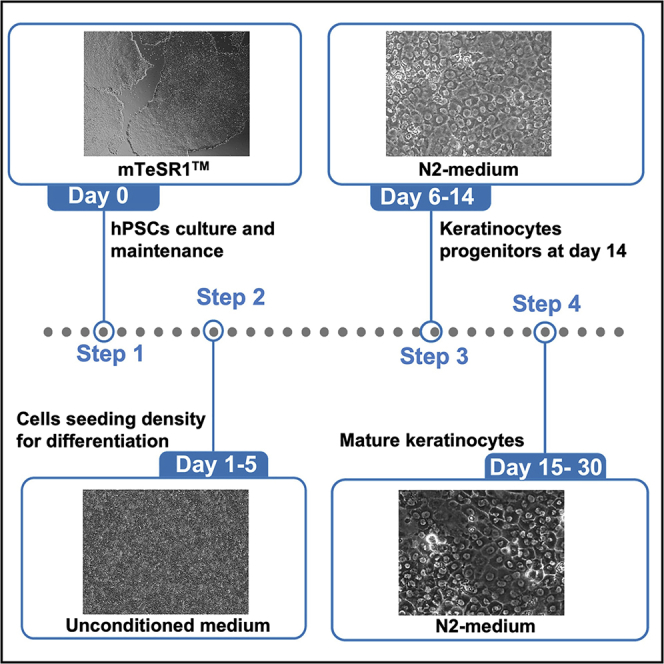

## Before you begin

For the execution of this protocol, we used human pluripotent stem cells (hPSCs), including embryonic (ESCs) and induced pluripotent stem cells (iPSCs). The cells were cultured and maintained in mTeSR1 medium in a humified 37°C incubator and 5% CO2. The cultured media, solutions, and matrix-coated plates were prepared before initiating the differentiation protocol. The growth factors and small molecules were added to the medium before use. Prior to the beginning of differentiation protocol, prepare media, solutions, and matrix-coated culture dishes. All media should be brought to 37°C prior to adding on the cells.

### Geltrex coating

1. Thaw Geltrex at 4°C for 24 h.
2. Dilute Geltrex in cold knockout DMEM/F12 as per dilution factor mentioned on the datasheet (final concentration of 100 mg/mL).
3. Add the diluted Geltrex to the dish to cover the entire surface.
4. Incubate at 37°C for at least 60 min. The coated dish can be store at 4°C and is stable for two weeks.

## Key resources table

**Table:** 

REAGENT or RESOURCE	SOURCE	IDENTIFIER
**Antibodies**		

Mouse anti-p63	Abcam	Ab735
Mouse anti-cytokeratin 1	Abcam	Ab81623
Mouse anti-cytokeratin 14	Thermo Fisher Scientific	MAS-11599
Rabbit anti-cytokeratin 18	Abcam	Ab181597
Mouse anti-cytokeratin 19	Chemicon	CBL198
Rabbit anti-Involucrin	Abcam	Ab53112
Rabbiti anti-Loricrin	Abcam	Ab85679
Rabbit anti -E-Cadherin	Cell Signaling	24E10
Rabbit anti-Filaggrin	Abcam	Ab81468
Rabbit anti-Laminin	Abcam	Ab14509
Mouse anti-Actin	Santa Cruz	Sc-47778
Donkey anti-rabbit, Alexa Fluor 488 (1:500 dilution)	Invitrogen	A21206
Goat anti-mouse, Alexa Fluor 488 (1:500 dilution)	Invitrogen	A21202
Donkey anti-rabbit, Alexa Fluor 594 (1:500 dilution)	Invitrogen	A10042
Donkey anti-mouse, Alexa Fluor 594 (1:500 dilution)	Invitrogen	A10037

**Chemicals, peptides, and recombinant proteins**		

DMEM/F12	Thermo Fisher Scientific	Cat#1260012
Knockout serum replacement medium	Thermo Fisher Scientific	Cat#10828010
N2-supplement	Thermo Fisher Scientific	Cat#17502048
mTeSR1	STEMCELL Technologies	Cat#85850
L-Glutamine	Thermo Fisher Scientific	Cat#25030081
Non-essential amino acids	Thermo Fisher Scientific	Cat#11140050
β-Mercaptoethanol	Thermo Fisher Scientific	Cat#21985023
RelesR	STEMCELL Technologies	Cat#05873
TrypLE Express Enzyme	Thermo Fisher Scientific	Cat#12604013
Penicillin/Streptomycin	Thermo Fisher Scientific	Cat#15140152
Retinoic acid	Sigma-Aldrich	Cat#R2625
BMP4	Thermo Fisher Scientific	Cat#PHC9531
EGF	Thermo Fisher Scientific	Cat#PHG0311
Matrigel hESC-Qualified Matrix	Gibco	Cat#A14133
Y-27632 dihydrochloride (Rock Inhibitor)	Cayman Chemical	Cat#10005583
Hoechst 33258	Invitrogen	Cat#H3569
CaCl2	Sigma-Aldrich	Cat#746495
Paraformaldehyde	Santa Cruz Biotechnology	Cat#Sc-281692
Bovine serum albumin (BSA)	Sigma-Aldrich	Cat#A7030

**Experimental models: Cell lines**		

hESCs line H1	WiCell Research Institute, Inc.	WA01
Ctr1-iPSCs	iPSC line generated in our lab from healthy individual	QBRIi001-A
Ctr2-iPSCs	iPSC line generated in our lab from healthy individual	QBRIi002-A
PsO1-iPSCs-C1	iPSC line generated in our lab from a patient with psoriasis	QBRIi005-A

**Oligonucleotides**		

OCT4: Forward (GACAGGGGGAGGG GAGGAGCTAGG); Reverse (CTTCCCT CCAACCAGTTGCCCCAAAC)	Integrated DNA Technologies	N/A
Nanog: Forward (CATGAGTGTGG ATCCAGCTTG); Reverse (CCTGAAT AAGCAGATCCATGG)	Integrated DNA Technologies	N/A
KRT18: Forward (GTACTGGTCTC AGCAGATTG); Reverse (CTGGCC TTCAGATTTCTCAT)	Integrated DNA Technologies	N/A
P63: Forward (TTCGGACAGTACA AAGAAC); Reverse (CCCTCACTG GTAAGTATAAC)	Integrated DNA Technologies	N/A

**Other**		

6-well plate	Eppendorf	Cat#0030720113
12-well plate	Eppendorf	Cat#0030721110
24-well plate	Eppendorf	Cat#0030722116

## Materials and equipment

**Table:** Differentiation medium composition

Unconditioned medium (UCM)	Final concentration	Amount
DMEM/F12	N/A	411 mL
Knockout Serum	15%	75 mL
L-Glutamine	1×	5 mL
NEAA	1×	5 mL
2-Mercaptoethanol	0.1 mM	0.9 mL
Penicillin/Streptomycin	0.5×	2.5 mL

• 50 mL aliquots in conical tubes can be used.
• The media can be stored at 4°C for up to 30 days.

**Table:** 

N2-medium	Final concentration	Amount
DMEM/F12	N/A	482 mL
N2-supplement	1×	5 mL
L-Glutamine	1×	5 mL
NEAA	1×	5 mL
2-Mercaptoethanol	0.05 mM	0.045 mL
Penicillin/Streptomycin	0.5×	2.5 mL

• 50 mL aliquots in conical tubes can be used.
• The media can be stored at 4°C for up to 30 days.

## Step-by-step method details

### Differentiation of pluripotent stem cells into keratinocyte progenitors

**Timing: 2weeks**

This section provides detailed description of obtaining keratinocytes from hPSCs. The first stage is the differentiation of hPSCs into keratinocyte progenitor-like cells by using retinoic acid (RA) to promote ectodermal fate (Bain et al., 1995) and bone morphogenetic protein 4 (BMP4) to block neural fate (Gambaro et al., 2006), which together can induce epithelial differentiation from hPSCs.

***Note:*** Ensure the quality of hESCs before starting the differentiation by checking the cell morphology and expression of key pluripotency markers, such as OCT4, NANOG, and SOX2. The cells should have a confirmed normal karyotype, free of differentiated cells, and mycoplasma contamination.

1. On day 0, remove the culture medium from the cells and wash with 1 mL PBS to remove dead cells. Treat the cells with RelesR for 4–5 min at 37°C, then remove ReLeSR and detach the cells with media by gentle pipetting. Collect the cells in a 15 mL Falcon tube, centrifuge at 130 g for 5 min at 22°C and plate on Geltrex (1:100) coated plates as small clumps in mTeSR1 containing 10 μM Rock inhibitor. Split one confluent well of 6-well plate into two wells of a 6-well plate (1:2 ratio) to attain a confluency of 90–100% next day. The cells can be maintained in culture until they reach 90–100% confluency.
2. Remove the hESC medium and wash the cells with 1 mL of DMEM/F12 basal medium. Start the differentiation by adding 2.5 mL of unconditioned medium (UCM) supplemented with 1 μM RA and 20 ng/mL BMP4.
3. Continue to culture the cells for five days in UCM containing 1 μM RA and 20 ng/mL BMP4 with media change every day.
4. On day 5, treat the cells with ReLeSR for 4–5 min at 37°C, and then completely remove the dissociating reagent. Dissociate the cells with media and pipette gently to make small clumps of approximately 10–20 cells, centrifuge at 130 g for 5 min at 22°C, and plate the cells at 1:2 ratio onto Geltrex-coated plates. For RNA extraction and immunostaining, one well of 6-well is splitted into 4 wells of a 12-well plate and 3 wells of a 24-well plate. Add 10 μM Rock inhibitor during passaging the cells in addition to RA and BMP4.
5. Switch the media stepwise from UCM to N2 medium. On day 6, mix UCM and N2 medium at 1:1 ratio and add to the cells for stepwise transition. Change the media completely to N2 medium on day 7 and continue to treat the cells with RA and BMP4 until day 8 with media change every day.
6. On day 8, withdraw RA and BMP4 and add 10 ng/mL EGF to the N2 medium.
7. Continue to culture the cells until day 14 with media change every day to differentiate the cells into keratinocyte progenitors (Figure 1).

**Figure 1:**
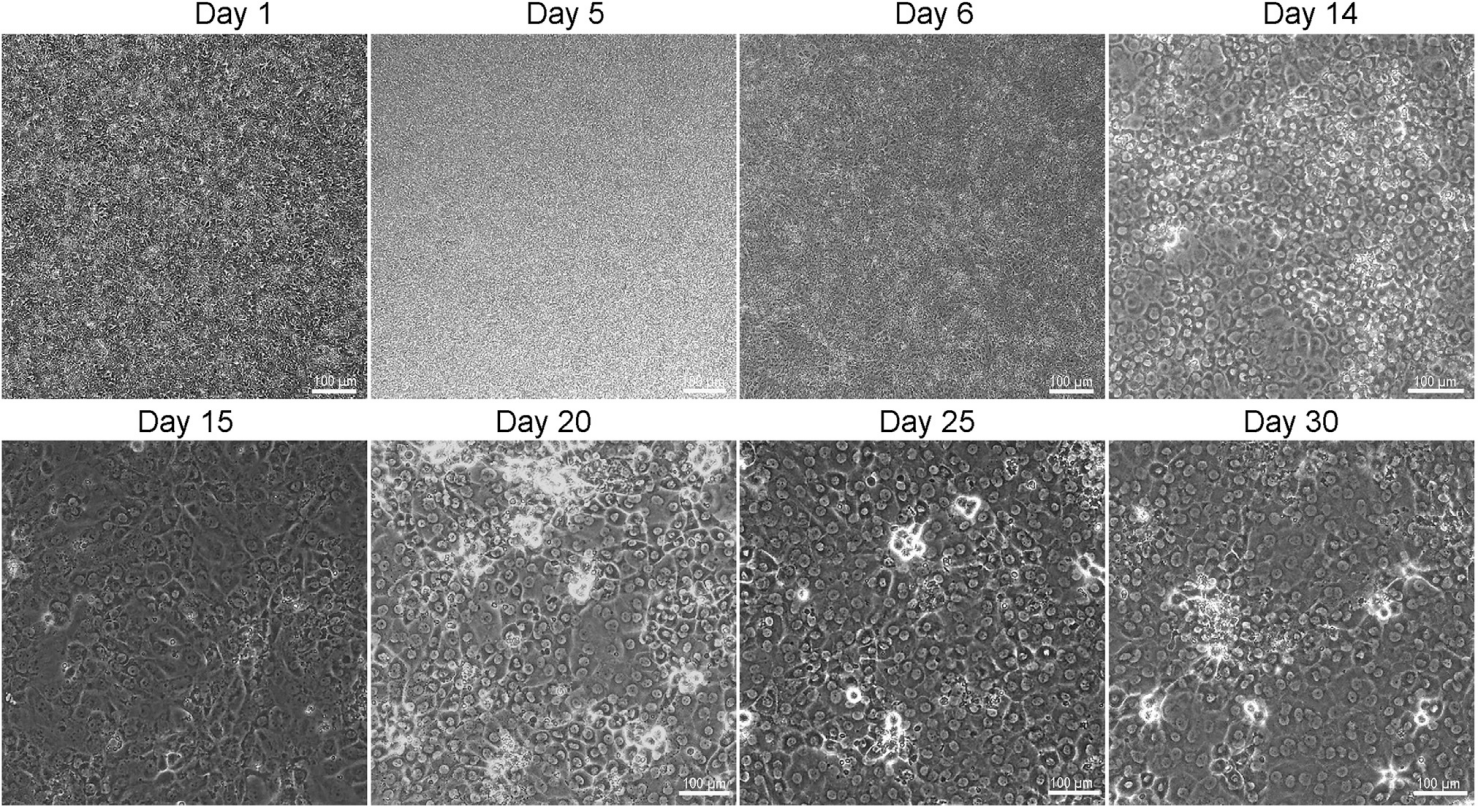
Differentiation of hPSCs into keratinocytes Bright field images showing different timepoints of differentiation into keratinocytes. Scale bar = 100 μm.
8. On day 14, check the keratinocyte progenitor cells for the expression of progenitor markers using RT-PCR and immunostaining (Figures 2A and 2B). Extract RNA from one well of a 12-well plate to check the expression of progenitor markers and use the cells grown in a 24-well plate for immunostaining.

**Figure 2:**
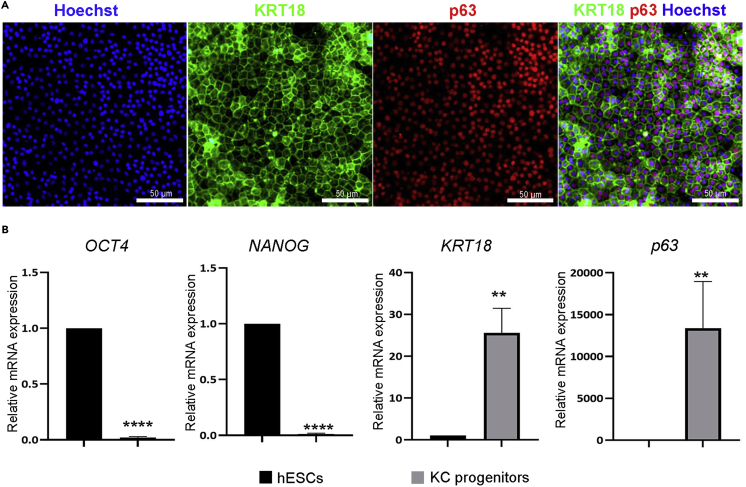
Expression of key markers in hPSC-derived keratinocyte progenitors (A) Immunostaining of keratinocyte progenitors at day 14 of differentiation with progenitor markers p63 (red) and KRT18 (green). The nuclei were stained with DAPI (blue). (B) qRT-PCR for pluripotency markers (*OCT4* and *NANOG*) and keratinocyte (KC) progenitor markers (*KRT18* and *p63*) in progenitor cells at day 14 of differentiation. Scale bar = 100 μm.

### Differentiation of keratinocyte progenitor cells into mature keratinocyte-like cells

**Timing: ∼2weeks**

9. On day 14, wash the keratinocyte progenitor cells with 1 mL PBS and treat with TrypLE for 8–10 min at 37°C. Gently remove the TrypLE and detach the cells with N2 medium. Following centrifugation at 130 g for 5 min at 22°C, plate the cells at 1:2 ratio onto Geltrex coated plate. Add 10 μM Rock inhibitor to the fresh media during passaging the cells.
10. On day 15, withdraw the Rock inhibitor and add N2 medium containing 10 ng/mL EGF.
11. Continue to grow the cells in N2 medium containing 10 ng/mL EGF for 8-days with media change every other day.
12. On day 23, add 1.2 mM CaCl2 to the N2 medium containing 10 ng/mL EGF for one week to induce maturation of keratinocytes and change media every other day.
13. On day 30, check the expression of mature keratinocyte markers using Western blotting and immunostaining. The mature keratinocytes can be passaged and kept in culture until day 45 (Figure 1).
14. For passaging keratinocytes, treat the cells with TrypLE for 4–5 min at 37°C. Gently remove the TrypLE and detach the cells with N2 medium. Following centrifugation at 130 g for 5 min at 22°C, culture the keratinocytes onto Geltrex coated plates in N2 medium containing 10 ng/mL EGF. Change the culture media every other day until day 45 (Figure 5).

**Figure 3:**
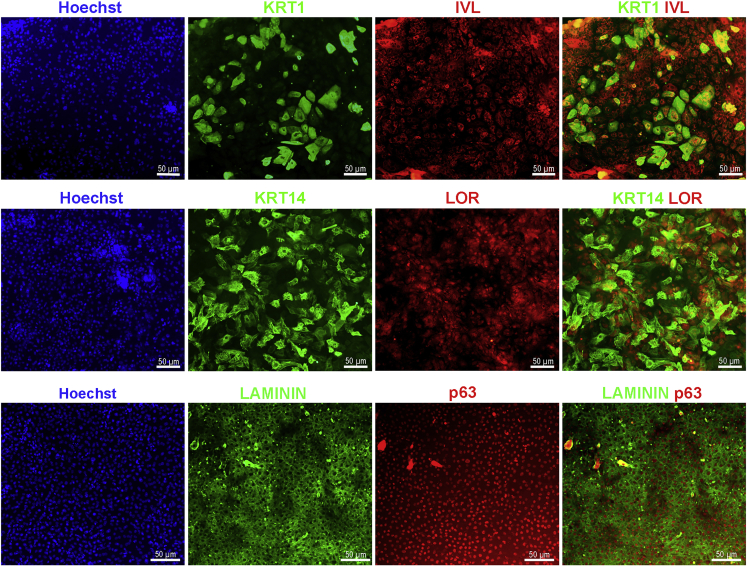
Immunostaining for mature keratinocytes at day 30 of differentiation Immunostaining images showing the co-expression of KRT1 (green) and Involucrin (red), KRT14 (green) and Loricin (red), or Laminin (green) and p63 (red). The nuclei were stained with DAPI (blue), Scale bar = 100 μm.

**Figure 4:**
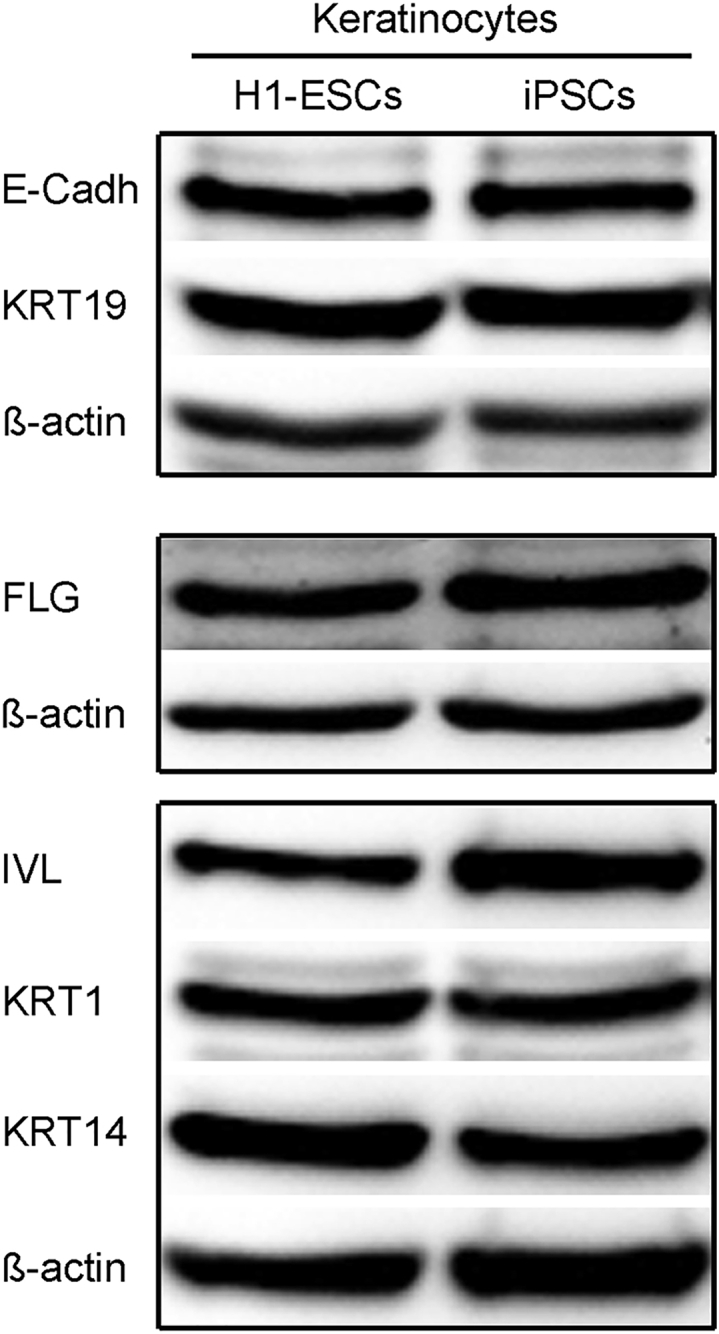
Western blot analysis for mature keratinocytes at day 30 of differentiation Western blotting showing the expression of key mature markers in keratinocytes derived from H1-ESCs and iPSCs at day 30 of differentiation.

**Figure 5:**
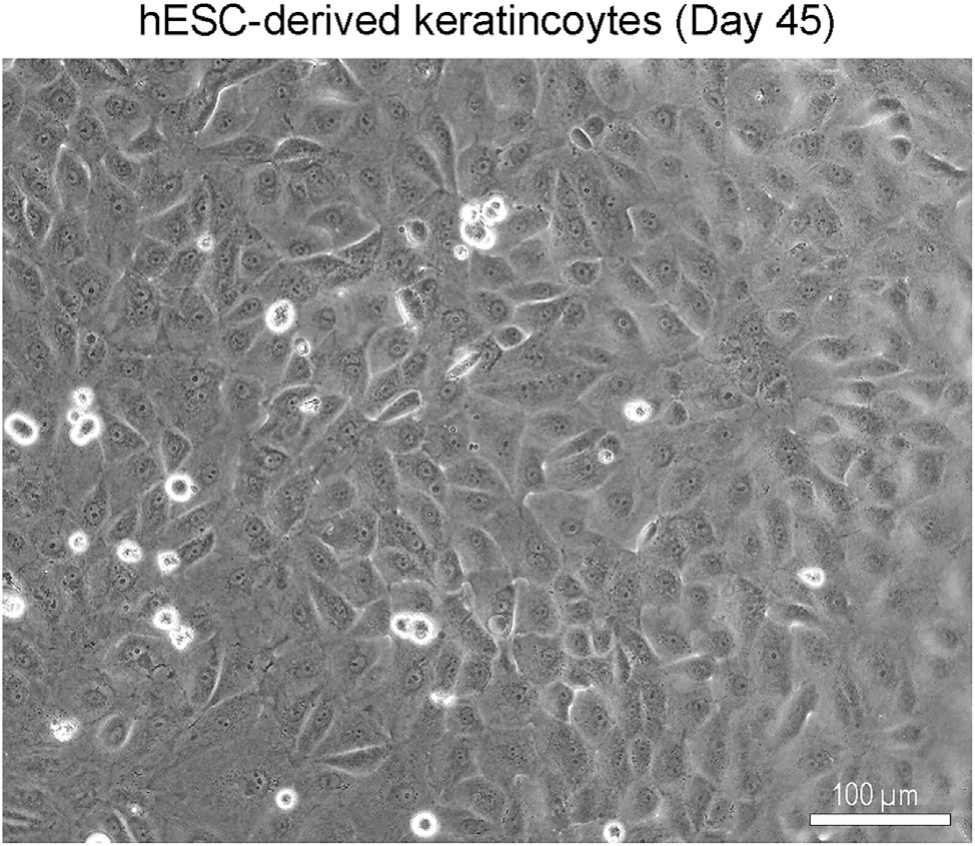
Keratinocytes at day 45 of differentiation Bright field image showing morphology of mature keratinocytes. Scale bar = 100 μm.

## Expected outcomes

Stem cell-derived keratinocytes offer a satisfactory human skin specific *in vitro* model to study disease pathogenesis, host-pathogen interaction, skin function, and drug screening. This protocol describes successful generation of mature keratinocyte-like cells from hPSCs that express markers of all skin layers as shown in Figures 2, 3, and 4. The hPSCs were differentiated using RA and BMP4, which are regulators of keratinocyte differentiation and proliferation and disrupt neuronal differentiation and induce ectodermal fate (Bilousova and Roop, 2013; Metallo et al., 2008). Treatment of hPSCs with RA and BMP4 induced the expression p63 and KRT18, resembling epithelial progenitors, while the expression of pluripotency markers (OCT4 and NANOG) was down-regulated (Figures 2A and 2B). Further differentiation and maturation of progenitors was achieved with calcium, which is the major regulator of keratinocyte differentiation *in vitro* and *in vivo*. A calcium gradient within the epidermis promotes the sequential differentiation of keratinocytes as they traverse the different layers of the epidermis to form the permeability barrier of the stratum corneum (Bikle et al., 2012). The hPSC-derived mature keratinocyte-like cells express markers of stratum basale (p63 and KRT14), stratum spinosum (KRT1), stratum granulosum (FILAGGRIN), and stratum corneum (LORICRIN and INVOLUCRIN) (Eichner et al., 1986; Metallo et al., 2010; Sebastiano et al., 2014) (Figures 3 and 4).

## Limitations

This protocol has been tested for the generation of keratinocyte-like cells in 2D culture condition using H1-hESCs and iPSCs derived from healthy controls and patients with psoriasis (Ali et al., 2020). However, the reproducibility of the protocol may vary with other hPSC lines and may need initial adjustment of passaging and cell density. The keratinocyte-like cells produced using this protocol express specific markers for mature keratinocytes; however, further characterization of these cells would be necessary by comparing with primary keratinocytes. Furthermore, the use of this protocol for the preparation of 3D iPSC-derived skin organoid needs to be optimized.

## Troubleshooting

### Problem 1

hPSC spontaneous differentiation and cell death (step 1).

### Potential solution

To overcome spontaneous differentiation and cell death, hPSCs should not be allowed to overgrow and passaging frequency needs to be optimized depending on the cell type. Aliquot the media containing supplements and store at −20°C to keep the activity of the growth factors. Use fresh culture media and change the media daily.

### Problem 2

Keratinocyte progenitor cells are difficult to dissociate (step 9).

### Potential solution

We observe the progenitor cells were hard to detach during passaging. To overcome this, treat the progenitor cells with TrypLE for 8–10 min at 37°C. Remove the dissociating solution, add fresh culture media and pipette the cells several times to detach from the surface. Centrifuge the cells at 130 g for 5 min at 22°C and plate at 1:2 ratio onto Geltrex-coated plate.

### Problem 3

Cell death during keratinocyte differentiation (steps 9–13).

### Potential solution

Seed the cells at high density and include 5 μM Rock inhibitor during passaging for 24 h.

### Problem 4

Low attachment of mature keratinocytes after passaging (step 14).

### Potential solution

Optimize treatment time with cell dissociation reagent and detach keratinocytes with the medium by gentle pipetting. Seed the cells at high density and add 5 μM Rock inhibitor during passaging for 24 h to increase cell survival rate.

## Resource availability

### Lead contact

Further information and requests for resources and reagents should be directed to and will be fulfilled by the lead contact, Essam M. Abdelalim (emohamed@hbku.edu.qa).

### Materials availability

Materials associated with this protocol are available upon request from the lead contact.

## Data Availability

This paper does not report datasets or original code.

## References

[bib1] Ali G., Elsayed A.K., Nandakumar M., Bashir M., Younis I., Abu Aqel Y., Memon B., Temanni R., Abubaker F., Taheri S., Abdelalim E.M. (2020). Keratinocytes derived from patient-specific induced pluripotent stem cells recapitulate the genetic signature of psoriasis disease. Stem Cell. Dev..

[bib2] Bain G., Kitchens D., Yao M., Huettner J.E., Gottlieb D.I. (1995). Embryonic stem cells express neuronal properties in vitro. Dev. Biol..

[bib3] Bikle D.D., Xie Z., Tu C.L. (2012). Calcium regulation of keratinocyte differentiation. Expet Rev. Endocrinol. Metabol..

[bib4] Bilousova G., Roop D.R. (2013). Generation of functional multipotent keratinocytes from mouse induced pluripotent stem cells. Methods Mol. Biol..

[bib5] Eichner R., Sun T.T., Aebi U. (1986). The role of keratin subfamilies and keratin pairs in the formation of human epidermal intermediate filaments. J. Cell Biol..

[bib6] Gambaro K., Aberdam E., Virolle T., Aberdam D., Rouleau M. (2006). BMP-4 induces a Smad-dependent apoptotic cell death of mouse embryonic stem cell-derived neural precursors. Cell Death Differ..

[bib7] Metallo C.M., Azarin S.M., Moses L.E., Ji L., de Pablo J.J., Palecek S.P. (2010). Human embryonic stem cell-derived keratinocytes exhibit an epidermal transcription program and undergo epithelial morphogenesis in engineered tissue constructs. Tissue Eng..

[bib8] Metallo C.M., Ji L., de Pablo J.J., Palecek S.P. (2008). Retinoic acid and bone morphogenetic protein signaling synergize to efficiently direct epithelial differentiation of human embryonic stem cells. Stem Cell..

[bib9] Sebastiano V., Zhen H.H., Haddad B., Derafshi B.H., Bashkirova E., Melo S.P., Wang P., Leung T.L., Siprashvili Z., Tichy A. (2014). Human COL7A1-corrected induced pluripotent stem cells for the treatment of recessive dystrophic epidermolysis bullosa. Sci. Transl. Med..

